# Metabolic Reprogramming: A Friend or Foe to Cancer Therapy?

**DOI:** 10.3390/cancers13133351

**Published:** 2021-07-03

**Authors:** Christopher McCann, Emma M. Kerr

**Affiliations:** Patrick G. Johnston Centre for Cancer Research, Queen’s University Belfast, 97 Lisburn Rd, BT9 7AE Belfast, Ireland; chris.mccann@qub.ac.uk

**Keywords:** drug resistance, cell death, cancer metabolism, mitochondria

## Abstract

**Simple Summary:**

Tumour cell metabolism is a dynamic and adaptive hallmark of cancer which enables cancer cells to survive, metastasise, proliferate and develop resistance to anti-cancer therapies. Here we review how metabolic reprogramming facilitates cancer cell survival, evasion of cell death processes such as apoptosis and facilitates the development of therapeutic resistance. We discuss how therapeutically-imposed metabolic dependencies can be rationally targeted to revert metabolic reprogramming from a supportive ‘friend’ to a fatal ‘foe’ of cancer cells.

**Abstract:**

Drug resistance is a major cause of cancer treatment failure, effectively driven by processes that promote escape from therapy-induced cell death. The mechanisms driving evasion of apoptosis have been widely studied across multiple cancer types, and have facilitated new and exciting therapeutic discoveries with the potential to improve cancer patient care. However, an increasing understanding of the crosstalk between cancer hallmarks has highlighted the complexity of the mechanisms of drug resistance, co-opting pathways outside of the canonical “cell death” machinery to facilitate cell survival in the face of cytotoxic stress. Rewiring of cellular metabolism is vital to drive and support increased proliferative demands in cancer cells, and recent discoveries in the field of cancer metabolism have uncovered a novel role for these programs in facilitating drug resistance. As a key organelle in both metabolic and apoptotic homeostasis, the mitochondria are at the forefront of these mechanisms of resistance, coordinating crosstalk in the event of cellular stress, and promoting cellular survival. Importantly, the appreciation of this role metabolism plays in the cytotoxic response to therapy, and the ability to profile metabolic adaptions in response to treatment, has encouraged new avenues of investigation into the potential of exploiting metabolic addictions to improve therapeutic efficacy and overcome drug resistance in cancer. Here, we review the role cancer metabolism can play in mediating drug resistance, and the exciting opportunities presented by imposed metabolic vulnerabilities.

## 1. Introduction

Changes to the cancer metabolome is not a new concept, nearly 100 years ago Otto Warburg reported that cancers had an enhanced avidity for glucose [1] and increased production of lactate [2], and these observations have since been confirmed across multiple cancer types [3] solidifying metabolic reprogramming as a key “Hallmarks of Cancer” [4,5]. More recently, refined methods of mapping metabolic programs within a tumour mass have demonstrated that multiple factors converge on dictating the metabolic landscape of a cancer, with driver oncogene [6], oncogenic levels [7], tissue of origin [6,8], spatial location within a tumour [9] and co-operating genetic alterations [10], all contributing to the metabolic programs underpinning cancer cell growth and spread.

Warburg originally postulated that the increase in glycolysis was to offset the metabolic deficit caused by damaged mitochondria in cancer cells [1]. Whilst indeed enhanced glycolytic flux and lactate production are key features of many cancer types, and a phenotype exploited to facilitate enhanced imaging techniques [11], we now appreciate that this is not simply due to mitochondrial failure. Instead, increased glycolytic flux provides required biosynthetic intermediates via branchpoints in the glycolytic cascade to support aberrant proliferation and redox buffering. To avoid a decreased TCA (Tricarboxylic Acid/Citric Acid) cycle flux as a result of negative feedback, an enhanced conversion of pyruvate to lactate prevents accumulation of NADH and ensures continued mitochondrial activity [5]. In fact, as the powerhouse of the cell, mitochondria play a key role in cancer development, contributing to this biosynthetic molecule and reducing equivalent production fundamentally required for enhanced cell growth [12]. Importantly, they also play a vital role in determining cell fate, by housing and interacting with a wide range of proteins involved in apoptosis and necrosis [13]. Thus, mitochondria are a key organelle, with a dynamic adaptive capacity to overcome cellular stress, that contributes to cancer development, progression and spread.

Drug resistance in cancer is a grave clinical concern, with tumour cells evolving to circumvent cytotoxic and cytostatic therapies [14], inevitably resulting in treatment failure and enabling the progression of disease. The mechanisms behind cancer cell drug resistance are varied and complex, often with multiple innate and acquired resistance strategies working cooperatively to enable cancer cell survival when facing a therapeutic insult ultimately designed to kill cancer cells and/or inhibit cell growth and proliferation. Put simply, resistance to treatment can be driven by tumour growth rate, tumour burden, tumour heterogeneity, physical barriers and drug efflux, undruggable driver mutations, immune evasion and therapeutic pressure selecting for resistant persister cells [14,15].

Although massive effort has been made to determine and target factors which drive drug resistance in cancer, many targeted therapies merely impose a selection pressure on a subset of cells within a heterogeneous tumour mass, effectively enabling expansion of resistant clones. Orthogonal treatment approaches targeting two seemingly independent pathways such as metabolism and apoptosis may offer a more powerful approach to improve therapeutic outcomes. Alternatively, and complimentary to this, Standard of Care (SoC) therapies, still a major clinical subset of the cancer therapy arsenal, may drive metabolic reprogramming specific to treatment and cancer subtype and thus converge ‘metabolically heterogenous’ tumour regions towards one common metabolic state, presenting novel imposed therapeutic vulnerabilities.

Within this review, we aim to address the interplay between metabolic reprogramming and cell death in cancers, how this metabolic flexibility can indeed promote therapeutic resistance, and explore the potential metabolic dependencies that may in fact enhance treatment response. Mechanisms of drug resistance are varied, diverse and intricate; hence we do not include an exhaustive list of resistance mechanisms in cancer (as these have been widely discussed elsewhere). The scope of this review is to focus on metabolic reprogramming as one of many complex mechanisms by which cancer cells overcome treatment-induced cell death.

### 1.1. Mitochondria—A Signalling Hub That Dictates the Balance between Life and Death

**Figure 1:**
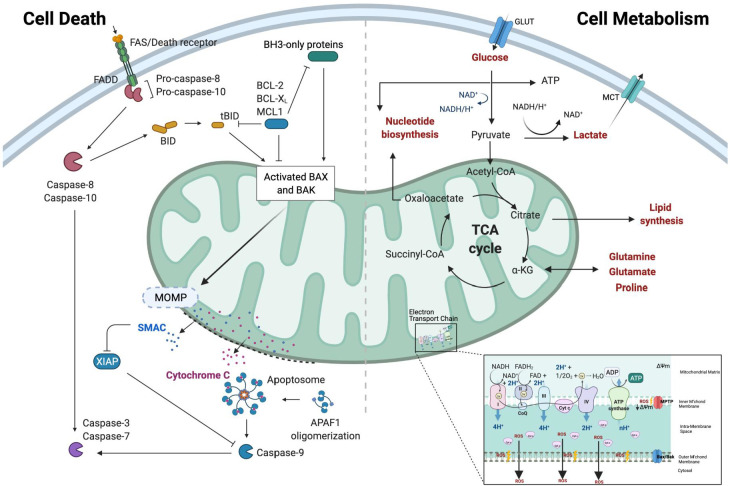
Mitochondria: A signalling hub between life and death. (**Left**) Death receptor activation by complementary ligand binding promotes the recruitment of the adaptor protein FADD and subsequent recruitment, homodimerization and activation of the initiator caspases-8 and 10, which in turn activate executioner caspases-3 and -7 directly to induce apoptosis; this is termed the extrinsic apoptotic pathway. Amplification of this death signal, via the mitochondria is mediated by Caspase-8 cleavage of BID. tBID then drives the translocation to and oligomerisation of BAX and BAK at the outer mitochondrial membrane (OMM), where insertion results in the formation of pores, driving MOMP. This process of intrinsic apoptosis is finely tuned by the activities of pro- and anti- BH3 family members such as MCL-1, BCL-2 and BCL-XL. MOMP results in the release of pro-apoptotic effector proteins including SMAC (Second Mitochondrial-derived Activator) and Cytochrome-c which inhibits anti-apoptotic proteins such as XIAP (X-linked Inhibitor of Apoptosis Protein) and promotes apoptosome formation, respectively, further activating the executioner caspases-3 and 7 and commitment to apoptosis. (**Right**) Cells uptake glucose via glucose transporters (GLUTs) where it enters the glycolytic cascade, a series of enzymatic reactions that ultimately yield ATP and NADH, terminating in pyruvate. High levels of pyruvate inhibit the metabolic process, and so pyruvate is either converted to Acetyl-CoA for further metabolism in the TCA Cycle or converted to lactate (glycolysis endpoint) which is exported from the cell into the extracellular space via MCT transporters. The TCA cycle and subsequent Electron Transport Chain (ETC) oxidises Acetyl-CoA to generate energy and biosynthetic intermediates to fuel lipid, amino acid and nucleotide synthesis. The ETC relies on an intact mitochondrial membrane potential to provide the proton gradient along which to transport electrons through each complex, ultimately resulting in the production of ATP through ATP Synthase. An important by-product from the ETC are reactive oxygen species (ROS) which can have widespread pro- and anti-tumourigenic properties. Abbreviations: *FADD:* Fas-Associated Death Domain, *tBID:* Truncated-BID, *SMAC:* Second Mitochondrial-derived Activator, *XIAP:* X-linked Inhibitor of Apoptosis Protein, *MCL-1:* Myeloid Cell Leukaemia-1, *BCL-2/BCL-XL* (B-Cell CLL/Lymphoma-2/XL), *MPTP:* Mitochondrial Permeability Transition Pore, *ATP:* Adenosine Triphosphate, *NADPH:* Nicotinamide Adenine Dinucleotide Phosphate Hydrogen, *ROS:* Reactive Oxygen Species.

Mitochondria lie at the intersection between cell metabolism, stress and death: acting as a sensor to cellular stress, an effector for engagement of changes in metabolic flux, and executioner of cell death through release of key engagement factors required for apoptosis induction [16]. The core apoptotic and metabolic processes associated with the mitochondria are depicted in Figure 1.

The mitochondrial genome is vital for the transcription of 13 proteins involved in oxidative metabolism and ATP production [17], and integrity of this unique organelle is required to maintain energy homeostasis and cellular survival [18]. The Kreb cycle (also known as the Citric Acid (TCA) cycle) [19], takes place within the mitochondrial matrix and is critical for the production of building blocks for biosynthesis to support proliferation, and the cofactors NADH and FADH_2_. These cofactors facilitate ATP production in the Electron Transport Chain (ETC), which occurs at the mitochondrial cristae. The fitness of the mitochondria to respond to cellular requirements and the modulation of metabolism in response to energetically demanding processes are key factors in the tumourigenic process, selected to facilitate escape from metabolic bottlenecks imposed by both rapid proliferation and cellular stresses, including those imposed by anti-cancer therapeutics. The coordination of metabolic adaption, and the balance between managing energy deficit and organelle integrity, is vital to ensuring continued cellular viability and inhibiting apoptosis. The AMP-activated protein kinase (AMPK), activated in low energy conditions, decreases ATP consumption and increases ATP production via the coordination of multiple metabolic signals. Recently, it has also been shown to be an intrinsic sensor of metabolic stress, critical for mitochondrial “health” and regulating fusion and fission [20] and is implicated in cell death processes such as apoptosis and autophagy [21]. In the last decade, researchers have gained a new appreciation for mitochondria as a signalling organelle that communicates with the rest of the cell, and neighbouring cells. Reactive Oxygen Species (ROS) generated as a by-product of mitochondrial metabolism were originally considered cytotoxic, but demonstrably can regulate cell growth and adaption to cellular stress [22]. In addition, the release of TCA cycle intermediates from the mitochondria can result in epigenetic modifications, and distinct transcriptional responses [23,24,25]. Furthermore, the release of mitochondrial DNA (mtDNA) upon loss of membrane integrity can also trigger an inflammatory response [26] (mtDNA discussed in further detail in Section 1.2). Thus, mitochondria are an important signalling hub, coordinating integration of numerous stress responses in cells, and vital to cancer cell survival and progression.

However, should damage or stress signals reach critical levels, the depolarisation of the mitochondrial membrane and subsequent release of cytochrome-c to the cytoplasm triggers activation of the intrinsic apoptotic pathway. The Bcl2 family of proteins are critical to control this depolarisation, with oligomerisation and insertion of Bax/Bak proteins into the outer mitochondrial membrane resulting in mitochondrial outer membrane permeabilisation (MOMP) [27]. A cell’s capacity to undergo MOMP is determined by its relative proportions of Bcl2 family members, with ‘activator’ proteins such as Bid and Bim activating the pro-apoptotic effectors Bax and Bak. Bax and Bak can be inhibited by anti-apoptotic proteins including Bcl-2 and MCL-1, which can be sequestered and inhibited by ‘sensitiser’ BH3 only proteins such as PUMA and NOXA [28]. The activity of these Bcl2 family members is finely tuned and ultimately linked to the mitochondria, hence any disruption to the expression of these proteins will have a significant impact on apoptotic propensity and mitochondrial integrity. Recent findings suggest non-apoptotic roles for some Bcl2 family members, such as MCL-1 which exists as distinct isoforms; one of which resides on the outer mitochondrial membrane to inhibit apoptosis and another mitochondrial matrix associated isoform which is required for optimal Oxidative Phosphorylation and ATP production [29].

MOMP, often considered the apoptotic ‘point of no return’, is characterised by the formation of pores in the outer mitochondrial membrane, which facilitates the release of the pro-apoptotic proteins SMAC/DIABLO and cytochrome-c into the cytoplasm. However, there is now growing evidence that MOMP may not be a terminal event in all cells, and the concepts of iMOMP (Incomplete MOMP—when MOMP occurs in some but not all mitochondria in the cell, and caspase activity is inhibited) and miniMOMP (Minority MOMP—when MOMP occurs in relatively few mitochondria and results in sub-lethal caspase activation) have been proposed [30]. The consequences of limited MOMP can be far-reaching and further transformative for the cell as a result of sub-lethal caspase activation and endonucleases inducing DNA double-strand breaks (DSBs), often in the absence of any death-causing stimulus [31]. Persistent low-level cytochrome-c “leakage” has been shown to induce DSBs through caspase-mediated endonuclease activation, these DSBs then recruit ATM where it is then phosphorylated and can activate downstream effectors to repair the damage or induce apoptosis [32]. Intriguingly, Liu et al., reported that ATM activated as a result of sub-optimal caspase activity via this mechanism can induce NFκB- and STAT3-mediated pro-survival signalling, resulting in a pro-tumourigenic and hyperproliferative stem-like phenotype [32]. Endonuclease-G is typically restricted to the mitochondria, until an apoptotic stimulus or loss of mitochondrial membrane potential prompts its translocation to the nucleus where it performs its DNA cleaving role [33]. Thus, mitochondrial integrity is essential to maintain genome stability and prevent the pro-tumourigenic effects of DNA damage.

**Figure 2:**
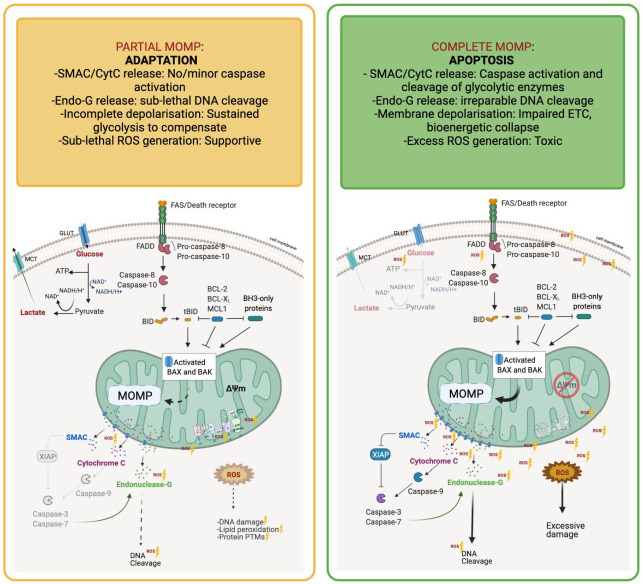
Incomplete MOMP promotes metabolic mechanisms of drug resistance. Mitochondrial Outer Membrane Permeabilisation (MOMP), when complete, results in SMAC and Cytochrome-C release and effective executioner caspase activation, promoting efficient induction of apoptosis. Depolarisation of the mitochondria result in the failure of the ETC and metabolic crisis. Caspases activate Endonuclease-G to facilitate irreparable DNA cleavage, and cleave core glycolytic enzymes, eliminating the ability for glycolysis to compensate for loss of ETC activity resulting in total bioenergetic collapse and toxic ROS generation. Together, this promotes effective cancer cell death. Partial (or incomplete) MOMP (iMOMP) facilitates dampened apoptosis engagement by the mitochondria, limiting caspase activity, and allowing cells to adapt to low-level mitochondrial damage. SMAC and Cytochrome-C are released, yet executioner caspases are not activated. Limited membrane depolarisation occurs, leading to sub-optimal ETC activity and associated sub-lethal (and protumourigeneic) ROS production. Glycolysis remains intact and compensates for the depletion in ETC processes. Thus, partial MOMP facilitates resistance to cell death and metabolic catastrophe in cancer cells, promoting therapy failure.

Cytochrome-c release during MOMP is thought to be biphasic; the release of a ‘loosely bound’ pool occurs first, with an amplification loop and mitochondrial cristae remodelling then triggering release of the more tightly bound pool [34,35]. The loosely bound ‘soluble’ cytochrome-c pool is that which is responsible for electron transport and ROS management, i.e., serves a critical role in mitochondrial oxidative phosphorylation and ATP generation, but interestingly these processes are not significantly interrupted until the second wave of cytochrome-c is released, suggesting metabolic compensation is possible during incomplete MOMP. The ‘tightly bound’ pool also interacts with cardiolipin (CL), facilitating cytochrome-c association with mitochondrial membranes. Amplification following the first wave can promote CL oxidation and subsequent dissociation from cytochrome-c, caspase activation and resultant cleavage of essential components of ETC complexes, which potentiates further ROS generation and respiratory collapse [34]. Ricci et al., revealed that following cytochrome-c release caspase-3 cleaves the p75 subunit (NDUFS1) of Complex I, resulting in further loss of mitochondrial membrane potential and mitochondrial integrity, production of ROS, plasma membrane damage and a fatal drop in ATP levels [36]. Therefore, levels of cytochrome-c release will dictate not only the levels of oxidative metabolism and ATP generation, but also apoptotic engagement, demonstrating that levels of MOMP are vital in modulating balance of metabolic and apoptotic responses to cellular stress. The processes and consequences of partial and complete MOMP are demonstrated in Figure 2.

An abundant by-product of mitochondrial respiration is the superoxide radical, O_2_^•^^−^, produced when electron donors proximal to the ETC machinery reduce O_2_ to produce this potentially harmful free-radical. The production of O_2_^•^^−^ is determined by numerous conditions, including enzyme abundance in the mitochondria, proportions of electron carriers and tissue-specific physiological oxygen availability [37]; hence any disruption to mitochondrial states, for example during cancer development or treatment, could have profound impact on free-radical production. The O_2_^•^^−^ can dismutate to form the reactive oxygen species H_2_O_2_, which can be degraded to H_2_O by glutathione peroxidases or scavenged by cellular antioxidants [37]. If cellular ROS buffering capabilities are overwhelmed, irreversible damage to lipids, proteins and nucleic acids will ensue, which may ultimately result in cell death or further oncogenic transformation. Of the known ‘targets’ for ROS-induced damage (mainly through post-translational modifications and/or HIF1α) are key metabolic enzymes such as PKM2, LDH and PFK1, which could further perpetuate the metabolic reprogramming often seen in highly oxidative tumours. Hence it is unsurprising that many cancer cells enrich both their oxidative metabolism and antioxidant management systems in tandem to evade cell death following treatment [38,39].

Therefore, metabolic and apoptotic cellular stress responses are intrinsically linked and coordinated through the mitochondria to facilitate cellular survival or cell death engagement. As such, altered cancer metabolism has the potential to act as both a *Friend* and *Foe* to anti-cancer therapies, where the ultimate goal is to induce cell death.

### 1.2. Driving Enhanced Cancer Growth Simultaneously Promotes Resistance to Death

As cells divide, the mitochondria play a key role in providing cellular substrates and energy necessary to facilitate growth. Often, the acquisition of oncogenic mutations or loss of tumour suppressor genes are responsible for driving this transformation process, and place a significant stress on the mitochondrial programs within a cell—the energetics must adapt to facilitate cell growth, and the anti-apoptotic machinery must be engaged to allow time for the cells to overcome this imposed bottleneck to growth. Thus, communication within the mitochondria is intrinsically linked, and often these cancer driver mutations will simultaneously result in differential metabolic and apoptotic programs [40].

Some of the most commonly altered genes in human cancer, *TP53*, *KRAS*, *PIK3CA* and *C-MYC* [41], have robust links with metabolic reprogramming and evasion of apoptosis when aberrantly expressed. *TP53*, the most commonly mutated gene across all cancers [41], is known to transcribe a number of pro-apoptotic genes such as PUMA, Noxa and Bax [42] whilst also inducing the transcription of a host of metabolic target genes [43]. The roles and functions of p53 are complex and often context-dependent, with some anticancer therapies shown to induce p53 mediated p21 upregulation and cellular senescence, and in some instances where sub-threshold DNA damage is inflicted this can induce a p53/p21 mediated anti-apoptotic response [44]. The metabolic demands of ‘healthy’, senescent and apoptotic cells are inherently different and p53 therefore plays a significant role in orchestrating these distinct phenotypes and subsequent metabolic demands. The p53 target gene *TIGAR* (*T*p53-*I*nduced *G*lycolysis and *A*poptosis *R*egulator) is a prime example of how p53 mediates direct crosstalk between metabolism and apoptotic signalling. TIGAR mimics the activity of the enzyme PFK-2/FBPase-2 and protects against ROS-induced apoptosis through enabling the accumulation of G6P as a result of PFK-1 inhibition; G6P is then shunted into the Pentose Phosphate Pathway (PPP) resulting in the generation of ROS scavenger, glutathione (GSH) [45]. Mutations in p53 can also have significant impact on core metabolic pathways and exacerbate the effects of lost tumour suppressor function. p53 is known to positively and negatively regulate core components of central carbon (glycolysis, TCA cycle and oxidative phosphorylation), lipid and the pentose phosphate pathway (for nucleic acid biosynthesis) via transcriptional and non-transcriptional mechanisms [43]. Loss of p53 is widely considered to facilitate unchecked tumour growth and contribute to poor prognoses, however the presence of distinct p53 mutant isoforms has been shown to support adaptation to nutrient deprivation through upregulating antioxidant defence systems—demonstrating a clear gain-of-function in metabolic remodelling and enhancing cancer cell survival [46,47]. The complexities of p53 regulating metabolic reprogramming are further compounded by tissue-specific differences in metabolic programmes [48]. Breast and lung cancer are both considered to be somewhat p53 driven [49] but show vastly different dependencies on lipid metabolism for tumour growth [50,51,52]. Hence p53 status should not be considered in isolation but must be examined in the wider context alongside other driver mutations and tissue metabolic demands.

*KRAS* mutations, common in lung, colorectal and pancreatic cancers, have been linked to many altered metabolic programs, including altered glucose [53], glutamine [54] and amino acid metabolism [8], ROS management [55], changes in autophagy [56] and extracellular macronutrient scavenging [57]. Again however, the context of *Kras* mutation and signalling matters—exemplified clearly by the differential role glutamine metabolism plays in *Kras* mutant lung tumours compared to colorectal or pancreatic [10,54,58,59]. In addition, the level of oncogenic signalling matters, with *Kras* mutant copy gains dictating enhanced glucose and glutathione dependencies in lung cancer models [7]. *KRAS* has also been linked significantly to driving resistance to apoptosis through the modulation of key apoptotic gene expression [60], and by enhancing pro-survival signalling via ERK/PI3K networks [61,62]. Downstream effectors of RAS, namely RALB, have been directly linked with death receptor (DR5) trafficking to the plasma membrane, and subsequent caspase-8 activation; exemplifying Ras’ indirect role in regulating apoptosis [63]. Mutant *Kras* has also been shown to drive the expression of apoptotic genes such as BCL-XL [64] and simultaneously increase the expression of core antioxidant enzymes through the activity of the NRF2 transcription factor [65], both of which have a profound impact on treatment response. Oh et al., observed a synergistic effect of targeting both BCL-XL and antioxidant defence systems in *Kras* mutant colorectal cancer [66], demonstrating the potential for targeting the multifaceted cell-protective roles for *Kras* in cancer.

*PIK3CA* is another gene frequently mutated/altered in human cancers [41] and, by dictating expression of the catalytic subunit of PI3K, contributes significantly to a number of cellular pathways that regulate cell growth, motility, metabolism and survival [67]. With links to mTOR, Akt and cMyc, it comes as no surprise that *PIK3CA* mutations can influence metabolic programs in a number of cancer types [67]. In colorectal cancer, it dictates glutamine dependency by modulating expression of glutamate pyruvate transaminase 2 (GPT2), enhancing the conversion of glutamate to aKG, and facilitating increased TCA utility [68]. In *PI3KCA* mutant breast cancers, an enhanced arachidonic acid metabolism driven via mTOR signalling, and increased lipid metabolism via SREBP1 signalling, were recently uncovered using REIMS technology across multiple sample types [69]. Importantly, PI3K also engages with a number of apoptotic pathways particularly via its interaction with Akt [70], and itself is a major contributor to the pro-survival stress response in cancer cells, driving increased drug resistance in many cancer types (elegantly reviewed by Liu et al. [71]).

The MYC family of transcription factors, deregulated in almost 70% of human cancers [41], also possess both apoptotic and metabolic genes in their target-gene repertoire [72,73]. Myc plays an important role in modulating cellular metabolism via controlling the expression of many key glycolytic genes, and in regulating glutamine metabolism and ETC activity [74,75], however this is indeed context specific, with distinct MYC driven programs depending on tissue type and other extracellular stresses [6,76,77]. In addition, MYC plays a driving role in amino acid metabolism, regulating the uptake of essential amino acids via modulation of transporter expression [78] or the synthesis of amino acids through increased expression of key enzymes (e.g., BCAT1 [79]). Furthermore, MYC can modulate lipid metabolism by altered enzyme expression within the mevalonate (HMGCR [80]) or via conversion of citrate to Acetyl-CoA by ACLY [81]. MYC also plays a key role in modulating the death response in cancer cells. Vitally, the levels of MYC dictate its choice of pro-survival or pro-death target repertoire [82]—a little extra MYC drives pro-survival signalling, too much and it triggers a pro-death response [83]. As MYC is often amplified in cancers, concomitant increase in the antiapoptotic BCL2 family often accompanies high levels of MYC activity, facilitating survival in the face of too much MYC [84]. Furthermore, MYC levels can dictate apoptotic response to DNA damage via its engagement with p53 [85], or by modulating oxidative stress responses via expression of γ-glutamyl-cysteine synthetase [86]. Therefore, MYC not only plays a major role in modulating metabolic responses within a cell, but also dictates the level of apoptotic priming and response to cellular stress, making it an important player in emergence of drug resistance.

Genetic aberrations in lipid, carbohydrate and amino acid metabolism have been identified as the most common metabolic cancer drivers across many different cancer types [48,87,88]. Patients with a higher number of mutations and copy number gains in the associated metabolic genes for these processes had poorer survival outcomes than those with fewer metabolic gene alterations; demonstrating the significant impact deregulated metabolism has on response to treatment and disease initiation and progression [87]. IDH1 (encoding a key enzyme in the TCA cycle, Isocitrate Dehydrogenase) is one of the most commonly altered metabolic genes across all studied cancer types [87], and its mutation is so robustly linked with glioma development and progression that this discovery has fuelled the initiation of a number of large-scale Phase I/II clinical trials targeting IDH1 in this disease setting [89]. Similarly, alterations in Hexokinase (HK- the apex enzyme in glycolysis) family of genes are associated with poorer disease outcomes in a range of solid tumours and impaired response to a wide range of anti-cancer therapies in vitro [88]. Interestingly, mutations in mitochondrial DNA (mtDNA) impacting key OXPHOS component genes accumulate with age in colonic epithelia, and aid tumour progression in the context of tumour suppressor loss [90]. Importantly, and perhaps unsurprisingly, mtDNA mutations have also been shown to impact apoptotic susceptibility [91], with depletion or mutation of mtDNA increasing apoptosis induction in the presence and absence of drug treatment, and simultaneously having a significant impact on mitochondrial respiration [92]. These studies obviate the significant role genomic and/or transcriptomic driven metabolic reprogramming plays across a wide range of cancer types, and how targeting such alterations could prove a powerful strategy to improve patient outcomes.

Therefore, genetic drivers of cancer, such as those described above, have a key role to play in orchestrating the mitochondrial signalling networks to carefully support rapid cellular expansion, whilst preventing apoptotic engagement. Understanding the context of signalling downstream from these alterations is vital in defining their therapeutic relevance.

### 1.3. Adaptations to Cellular Stress Prevent Metabolic Catastrophe and Death

Enhanced proliferation in a developing cancer mass increases the nutrient requirements placed upon these rapidly dividing cells, to facilitate biosynthesis, ATP generation and redox buffering [93]. Often, enhanced growth factor signalling can facilitate increased nutrient uptake, but levels are also dictated by the environment in which a cancer is developing. Nutrients, delivered via the blood stream, can be limiting in a growing tumour mass until such times as an efficient network of vessels can be established. Nutrient stress is therefore thought to be fluid in a developing cancer, and the response to this nutrient deprivation, or indeed altered nutrient requirements, must be equally as flexible. Transcriptional control of transporters and key metabolic enzymes allow a cell to “fine tune” the metabolic programs in response to nutrient stress [94], with distinct transcription factors such as HIF1a (hypoxic stress), SREBP (lipid depletion) or ATF4 (amino acid deprivation) eliciting defined programs of adaption in a context dependent manner [94].

Low levels of O_2_ availability (termed hypoxia) impose a significant physiological stress on the tumour mass, and is often attributed as a cause of metabolic reprogramming in solid tumours [95]. A common feature of poorly vascularised tumours, hypoxia correlates with poor clinical outcomes [96]. The transcription factor hypoxia-inducible factor 1 (HIF1) is a major regulator of the metabolic response to hypoxia, altering glycolysis and mitochondrial metabolism in tandem to balance O_2_ demand with O_2_ availability [95]. HIF increases the expression of glycolytic genes, including glucose transporters (GLUT1, GLUT3) and many enzymes in the glycolytic cascade (HK, ENO, GAPDH, LDH) [97,98], which in turn results in increased glycolytic rates. Concurrently, HIF can also decrease glucose flux into the mitochondria via pyruvate, through increased expression of Pyruvate Dehydrogenase Kinase (PDK), which inhibits PDH-mediated conversion of pyruvate to Acetyl-CoA and TCA cycle entry [98]. HIF also targets proteins that regulate cell death, including a number of Bcl2 family members such as Bax, Bak, Bid, Noxa and Mcl1 [99], and the balance of these control cell survival as described above. Thus, induction of HIF has a major impact on the metabolic and apoptotic responses to cellular stress in cancer cells, including mediating therapeutic response [100]. More recently, HIF signalling has been associated with the process of epithelial to mesenchymal transition (EMT) through regulation of key genes that mediate membrane degradation (MMPs) and mesenchymal transformation (Vimentin, TGFa, cMET) [98]. This change in cellular phenotype has also been linked to a more “drug resistant” state [101]. Importantly, HIF expression is not only induced by low O_2_, but also in response to a number of oncogenic signals including *KRAS*, *TP53* and *PI3K* mutations. In addition, the accumulation of TCA cycle metabolites Fumarate and Succinate can also induce HIF expression via epigenetic modifications [25]. Thus, HIF is intrinsically linked to metabolic and apoptotic signalling in cancer, and facilitates cross-talk between these two major cancer hallmarks.

The mechanistic target of rapamycin (mTOR) signalling axis is a key player in modulating cellular metabolism. Originally described as a key regulator of protein synthesis, mTOR is also intrinsically linked to nutrient sensing and metabolic coordination (elegantly reviewed in [102]), facilitating cellular response to changing growth factor, oxygen, glucose and amino acid levels. mTOR, the major catalytic subunit of two distinct complexes, mTORC1 and mTORC2, is itself a serine/threonine protein kinase that facilitates a plethora of downstream signalling to control metabolism (mTORC1&2), protein synthesis and turnover (mTORC2), apoptosis (mTORC1), cell migration and morphology (mTORC1) [103]. Activation in mTOR signalling promotes increased metabolic flux and cell growth supporting cancer development progression and spread. In fact, mTOR signalling is enhanced across a number of cancer types [104], and can be regulated by a number of cancer-driving genetic alterations including *PIK3CA* and *KRAS* mutations or loss of the tumour suppressor *PTEN*. As such, mTOR in cancer is an attractive therapeutic target, and a number of strategies have been explored to effectively inhibit mTOR biology, including use of Rapamycin, the inhibitor of mTORC1 [105]. Importantly, the mTOR pathway is in turn regulated by AMPK, with activation of AMPK by cellular stresses such as nutrient deprivation or growth factor withdrawal resulting in mTOR inhibition, switching cells from anabolic to catabolic metabolism to help restore the deficit [106]. Thus, in a stressful evolving microenvironment like that of a growing cancer, mTOR plays a critical role in controlling the metabolic responses [107]. Importantly, mTOR also plays a critical role in modulating the balance between life and death of a cancer cell [108]. Enhanced mTOR signalling can promote a pro-survival response in cells, and modulate expression of a number of antiapoptotic proteins within the Bcl2 family, thus maintaining functional mitochondria [109,110]. However, in response to cellular stress (including treatment induced damage) the balance tips, and can elicit a pro-apoptotic response [105]. Indeed, upregulation of mTOR signalling has been demonstrated to promote resistance to anti-cancer therapies by enabling escape from cell death [111]. Interestingly, a number of strategies targeting mTOR and apoptotic priming concurrently have shown promise in improving anti-cancer therapeutic responses [112,113] to tip the balance of BH3 signalling towards a pro-apoptotic phenotype. Therefore, by navigating the cellular response to stress and the balance of metabolic and pro/anti-apoptotic signalling, the mTOR pathway acts as a key mediator of mitochondrial fitness, signalling and integrity; facilitating resistance and ultimately therapy failure. However, these metabolic dependencies on mTOR signalling result in an imposed vulnerability in cancer cells that may provide a unique axis to tip the balance away from *Foe* and towards a *Friend* of cancer therapy.

Cancer cells also exploit their metabolic plasticity in order to aid their survival and avoid death during metastasis [114] which imposes significant metabolic and physical stresses on the cell. Detachment from the extracellular matrix is an early event in the metastatic process which often results in the initiation of a form of cell death known as anoikis due to lack of pro-survival signalling [115]. Some metastatic cancer cells have developed metabolic adaptations to evade death by anoikis. Notably, cancer stem cells (CSCs) possess an innate ability to endure the demands imposed by the process of metastasis [116], and this has been proposed by some to be in part as a result of CSC metabolic reprogramming [117]. For example, pancreatic cancer stem cell populations were identified by Kim et al., to express higher levels and activity of the glycolysis-regulating enzyme Aldehyde Dehydrogenase 1 (ALDH1) [118], which aids their survival during metastasis and seeding in distant metastatic sites [119]. Furthermore, when detached, mammary epithelial cells have been reported to upregulate the expression of PDK4 (Pyruvate Dehydrogenase Kinase 4) thus inhibiting PDH and restricting the flux of glucose-derived carbon into mitochondrial oxidation. Such an adaptation reduces mitochondrial respiration and the oxidative stress associated therewith, meaning the detached cells can avoid anoikis induced by ROS accumulation [120]. Additionally, detachment of some cancer cell lines has been observed to promote upregulation of PDK4, and a subsequent suppression of PDH flux; resulting in a reduced proliferative capacity. Many cancers have relatively low PDK4 mRNA in comparison to normal tissues, presumably as a mechanism to negate its anti-proliferative effects [121]. These findings demonstrate the complexities and context dependence of metabolic reprogramming in cancer metastasis and expansion. Oncogenic KRAS can simultaneously inhibit anoikis and enhance glucose uptake with subsequent ATP generation when cells detach from their ECM during metastasis [122]. Interestingly, metastasising melanoma cells can evade ferroptotic death by reprogramming fatty acid metabolism thus elevating levels of oleic acid and by increasing glutathione synthesis; thereby protecting them from oxidative stress and ferroptosis, aiding their survival and distant metastasis via the lymph vasculature [123]. Once seeded in their new metastatic niche the tumour cells must adapt their metabolic profile to fit the differing metabolic demands and nutrient availability of the new host tissue. That said, in a large scale in-silico study Gaude and Frezza revealed that suppression of OXPHOS genes was common in metastatic cells compared to the primary tumours from a range of anatomically and metabolically distinct primary sites, and such a gene expression signature was associated with poorer clinical outcomes [48].

The context in which metabolic studies are executed and interpreted is hugely important, with an ever-expanding array of complex and diverse 2D and 3D in vitro culture models and in-vivo models adding to the complexity and context specificity of deconvoluting the role of metabolic adaptation and treatment resistance in cancer models. The ability for 3D in vitro cultures to better recapitulate the pathophysiology of tumours in-situ has long been appreciated, however the capabilities and cost of 3D culture for high-throughput drug sensitivity screens is often prohibitive compared to traditional 2D cell culture. Therefore, more user-friendly and cost-effective 3D systems are needed to facilitate the transition to 3D culture screening [124]. A recent study comparing colorectal cancer (CRC) cell lines cultured as 2D, 3D and 3D cocultures with stromal cells revealed that each culture system imposed significant alterations in drug sensitivity, demonstrating the context specificity with which drug-sensitivity data must be interpreted [125]. Oxygen and nutrient gradients (among other cell-cell contact signalling pathways) within the 3D culture and in-vivo tumour setting can impose significant metabolic heterogeneity (concepts previously discussed above) within a tumour and select for more ‘metabolically robust’ and aggressive cell types capable of surviving in such inhospitable tumour microenvironments [124,126]. Metabolic stress imposed by 3D culture in p53-deficient CRC models leads to a transcriptional and functional metabolic shift towards mevalonate synthesis which promotes the production of ubiquinone and supports the TCA cycle. Importantly, this mevalonate/TCA cycle axis is much less pronounced in 2D models with p53 deficiency compared to their 3D counterparts, which demonstrates the interplay between genetics and culture conditions in modulating tumour cell metabolism [127]. Furthermore, encouraging results from parallel CRISPR screens in in vitro and in vivo pancreatic cancer models revealed that metabolic dependencies between 2D and 3D cultures were somewhat overlapping, whilst 3D cultures better recapitulated some distinct aspects of tumour metabolism not seen in 2D culture [128]. In vivo tumour metabolism is perhaps best recapitulated in vitro with organoids, which accurately replicate the genetic and functional characteristics of the tumour, albeit with the caveat that stromal and immune cell interactions are excluded [129,130]. Hence culture methods play a significant role in coordinating the cancer cell metabolic profile and drug sensitivities, therefore validation of metabolic adaptations in response to treatment across multiple in vitro and in vivo models provides the best opportunity to draw robust and cohesive conclusions to allow the most promising clinical targets to be identified.

### 1.4. Metabolites as Direct Mediators of Cancer Cell Death

Metabolic genes (such as HK2, PKM2 and FBP1) are also prone to epigenetic modification, which can result in more subtle real-time responses to various biochemical cues [131]. Metabolic products and intermediates have previously been reported to impose epigenetic changes on DNA and histones, leading to transcriptional changes and impacting DNA damage repair, which will conceivably impact baseline apoptotic susceptibility and response to DNA damaging therapies. Furthermore, epigenetic modification of core metabolic genes imposed by metabolites could potentially exacerbate or alleviate the metabolic rewiring characteristic of some cancer types [131]. Such ‘oncometabolites’ have also been shown to epigenetically impact the transcription of a host of genes related to cell death. For example, butyrate (a product in the TCA cycle) is often elevated in cancer cells and has been shown to inhibit HDAC activity in colonocytes [132] and cause the downregulation of some anti-apoptotic genes including Bcl-2 [133]. Similarly, pyruvate has proven ability to inhibit HDAC1/HDAC3 and induce apoptosis [134]; however, many cancers upregulate the LDH-A (lactate-deydrogenase-A) gene [135] to enhance the conversion of pyruvate to lactate and overcome these pro-apoptotic effects. In many respects the metabolite 2-hydroxyglutarate, an intermediate in the TCA cycle can be considered a ‘classical’ oncometabolite which exerts its pro-tumourigenic function through epigenetic effects, hypoxia regulation and immunosuppressive activity (expertly reviewed in [136]). Notably, silencing or inhibition of class-I HDACs have been observed to manipulate the expression of key players in both the intrinsic and extrinsic apoptotic pathways [137,138]. It has been reported that the transcriptional result of HDAC inhibition is generally pro-apoptotic and anti-proliferative [139] so cancer cells may have adapted mechanisms to counteract the HDAC-inhibitory activities of these oncometabolites which accumulate intracellularly and extracellularly.

Importantly, some nutrients/metabolites can directly regulate the expression and binding of certain BCL-2 family members [140], impacting apoptotic ‘priming’ [28] and the propensity of cells to undergo apoptosis. The anti-apoptotic BCL-2 family member MCL1 is reportedly more stable in highly glycolytic conditions due to the enhanced inhibitory phosphorylation of GSK-3α/3β; a kinase implicated in the degradation of MCL1. Thus, cells would be less apoptotically primed whilst in glucose deprived conditions, with reduced mTOR activity as a result of AMPK activation resulting in the inhibition of MCL1 translation and overall reduction of MCL1 levels [141], in turn lowering the apoptotic threshold. Zhao et al., reported that elevated glucose metabolism restricts p53-mediated induction of the pro-apoptotic protein PUMA and thus Bax-dependent apoptosis through MOMP [142]. In cells which lack the ability to induce MOMP, such as in the case of MCL-1 overexpression or dual Bax/Bak deficiency, glucose deprivation has been shown to induce caspase-8 dependent but death ligand-independent apoptosis [143]. Metabolic enzymes can also play a role in orchestrating apoptosis execution. For example, HK2 can inhibit apoptosis by binding to the N-terminus of VDAC1 (Voltage Dependent Anion Channel) on mitochondrial membranes, thus preventing cytochrome-c release and subsequent bioenergetic collapse and apoptosis [144]. Hence, glucose availability and metabolism, including enzymatic activity, can have significant impact on the apoptotic potential of cancer cells; meaning metabolic restriction has the potential to improve response to a range of cytotoxic therapies.

Apoptotic proteins have the potential to regulate distinct metabolic pathways. Mitochondrial integrity and metabolic function can also be controlled by BCL-2 family proteins; which sheds light on a non-canonical role for these ‘apoptosis proteins’. For example, the pro-apoptotic BH3-only protein BAD has been shown to interact with and activate glucokinase (GK) when phosphorylated, facilitating GK’s role as a glucose sensor and regulating entry into the glycolytic pathway. When dephosphorylated, BAD can no longer activate GK, which then liberates BAD to inhibit pro-survival BCL-2 family members and prime cells for apoptosis [145,146]. NOXA, another BH3-only protein, when phosphorylated by CDK5 (Cyclin-dependent Kinase 5) directs glucose-derived carbons from glycolysis into the pentose-phosphate pathway to drive nucleic acid synthesis, whilst its apoptotic function is simultaneously negated. Furthermore, MCL-1 and BCL-2 have been shown to serve a role in regulating mitochondrial respiration through facilitating the assembly of complexes I, III, IV [29] and the cytochrome-c oxidase activity of the electron transport chain [147], respectively.

Careful considerations must therefore be made prior to using BH3 mimetic drugs to determine the likely metabolic adaptations which arise in response to such ‘apoptosis priming’ therapies. These adaptations offer the opportunity to target apoptotic pathways concurrently with glucose metabolism pharmacologically or with nutrient restriction. Alternatively, and perhaps more clinically viable, is the use of SoC therapies to force specific metabolic dependencies which could be therapeutically targeted as a combination strategy.

### 1.5. Metabolism and Therapy Resistance: Exploiting Imposed Vulnerabilities to Improve Response

Cytotoxic therapies employed to ultimately induce cell death can also manipulate cell metabolism, either negating or potentiating the effect of these drugs. Metabolic reprogramming can in fact act as a driver of drug resistance and such adaptations are both treatment and site specific [48]. SoC treatments such as radio- or chemo- therapy are well reported to impose metabolic changes in cancer cells, and metabolic adaptation to treatment is widely observed to impact treatment response and patient outcomes [148]. An intriguing study by Lee et al., identified that chemo-resistant hepatocellular carcinoma cell lines use ATP produced by OXPHOS to drive drug-efflux pumps, and preferentially use glutamine rather than glucose to fuel this resistance mechanism [149]. Furthermore, high OXPHOS activity has been identified as a targetable mediator of chemo-resistance in pancreatic cancer [150]. Complimentary to this, chemo-resistant AML cells are highly dependent on OXPHOS processes for their persistence and repopulation [151], highlighting the crossover in metabolic reprogramming in both solid and haematological malignancies. Such observations provide strong evidence that cancer cells can exploit their metabolic plasticity and alternate between preferred fuel sources to drive intricate resistance mechanisms.

One mechanism of action of DNA damaging chemotherapies is to enhance ROS production. 5-Flurouracil (5-FU) enhances ROS production through various mechanisms including via Romo1 (Reactive Oxygen Species Modulator 1). Romo1 can induce tonic levels of ROS promoting pro-survival effects [39], and a simultaneous increase in antioxidants and the anti-apoptotic Bcl-2 [152]. 5-FU also induces ROS-dependent Src activation in colorectal tumours, leading to caspase-7 phosphorylation and apoptosis. Notably, the ability of 5-FU to induce apoptosis was significantly diminished with the addition of antioxidants; confirming a key role for ROS in 5-FU induced apoptosis [153]. Conversely, 5-FU also reportedly enhances CRC cell survival in high-glucose conditions as a result of enhanced glycolysis and a subsequent elevation in intracellular pyruvate, which acts as a ROS scavenger to protect against oxidative stress-induced death [154]. Thus, 5-FU treatment can improve the ability of cancer cells to tolerate oxidative stress and evade apoptosis induced by high levels of ROS. Cisplatin treatment increases mitochondrial mass and OXPHOS function in lung cancer cells [155] resulting in increased ROS generation. However, resistance to Cisplatin can reportedly be overcome by inhibiting OXPHOS with Metformin [155]. Similarly, targeting respiratory complex-I of the ETC with Phenformin overcomes chemo-resistance in pancreatic cancers with characteristically high OXPHOS activity [150]. Cisplatin can also promote glycolysis through stimulating PFKFB3 acetylation and retention in the cytoplasm where it carries out its role as a key glycolytic enzyme [156].

Glutamine metabolism and glutathione biosynthesis have also been shown to promote chemo-resistance in a range of cancer subtypes and genotypes, primarily but not exclusively owing to the antioxidant role of glutathione (GSH). Disruptions in the GSH:GSSG ratio, either through impaired GSH synthesis or a substantial increase in intracellular ROS are widely reported to dictate apoptotic sensitivities through various mechanisms [157]. Indeed, targeting glutamine/glutathione metabolism has been reported to overcome chemo-resistance across multiple cancers. Jagust et al., observed that chemo-resistant pancreatic cancer stem cells depend strongly on enhanced glutathione for protection against chemotherapy-induced cell death. Targeting GSH synthesis and recycling induced apoptosis alone and re-sensitised cells to SoC chemotherapy [158]. Intriguingly, chemo-resistant AML cells are stimulated to uptake more glutamine for glutathione synthesis following chemotherapy treatment, and targeting this therapeutically-imposed vulnerability significantly reverted the resistant phenotype [159]. Furthermore, it has recently been reported that cancer cell lines from a variety of tissue types bearing *RAS* mutations have significantly elevated NOX (NADPH-Oxidase) activity and GSH biosynthesis; contributing to simultaneously increased ROS production and scavenging capacity respectively [160]. *RAS* mutations are widely associated with poor prognosis and resistance to various SoC and targeted therapies. Dual targeting of both NOX and GSH selectively and synergistically killed cancer cells bearing oncogenic RAS, and unveiled their dependency on ROS buffering for survival [160].

Radiotherapy is also known to impose metabolic reprogramming across a wide range of cancer types through diverse and intriguing mechanisms [161]. Observations in HNSCC reveal that radioresistant cell lines harbour a greater number of mtDNA mutations, likely imposed by concomitant elevated levels of ROS and enhanced ATP production owed to elevated OXPHOS activity. Importantly, these findings are recapitulated in patients who respond poorly to radiotherapy which reflects the clinical implications of altered mitochondrial metabolic activity and treatment response [162]. It is likely that the mtDNA damage imposed by radiation results in ROS generation and a self-perpetuating loop of further DNA damage, mitochondrial reprogramming and ROS production [163,164]. Radiotherapy also alters STAT1, promoting increases in glycolytic, OXPHOS and TCA cycle genes which contribute significantly to the radioresistant phenotype [165]. CDK1 activation by radiotherapy also results in phosphorylation and activation of SIRT1, subsequently promoting enhanced ROS scavenging and ATP generation within the mitochondria, promoting radioresistance [166].

Targeted therapies such as the BH3-mimetic Venetoclax, designed to selectively inhibit the anti-apoptotic protein BCL-2, inhibits mitochondrial respiration and disrupts TCA cycle flux by causing mitochondrial deformity, initiation of the integrated stress response and activation of ATF4, possibly potentiating the pro-apoptotic mechanism of action and enhancing cell death [167]. Intriguingly, ATF4 has also been linked to the induction of both necrosis and apoptosis following either glucose deprivation or 2-Deoxyglucose treatment, respectively [168], demonstrating a potential mechanism through which impaired glucose acquisition kills.

**Figure 3:**
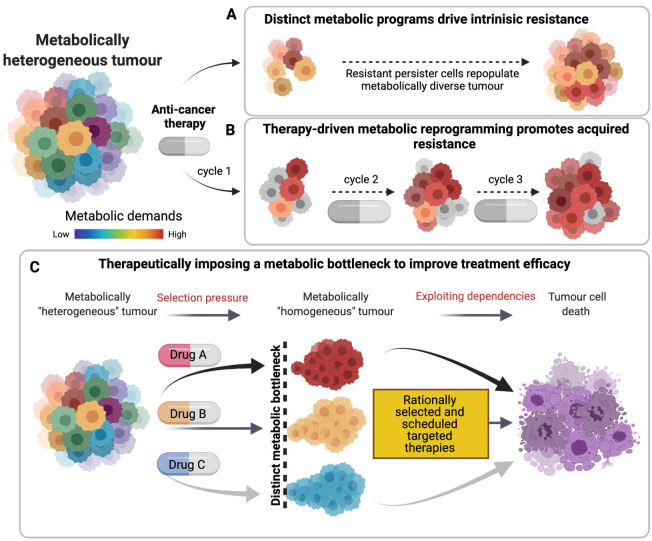
Tipping the metabolic balance from *Foe* to *Friend* of cancer therapy. (**A**) Intrinsically treatment-resistant cancer cells have distinct metabolic advantages to allow persistence and repopulation of metabolically diverse cell populations. (**B**) Treatment can enrich for more metabolically ‘fit’ cell populations, becoming purer with each treatment cycle and ultimately generating a metabolically homogenous tumour through acquired resistance. (**C**) Therapeutically induced metabolic dependencies (bottlenecks) can offer unique targets to improve treatment efficacy. It should be noted that the authors propose this model as so, a model, which does not necessarily depict all cancer types or treatment responses.

Therefore, anticancer therapies can modulate the metabolic responses in cancer cells, exacerbating or inhibiting the death responses, and so potentiating or limiting treatment efficacy in a context dependent manner. In turn, these imposed metabolic changes may present a novel opportunity for combination therapy approaches. Using therapy to drive heterogeneous tumours towards a distinct, more homogeneous metabolic dependency offers a therapeutic window to target a greater fraction of tumour cells efficaciously. This concept was eloquently demonstrated by the Scadden lab, describing how “Induction of a timed metabolic collapse was used to overcome chemo-resistance” in acute myeloid leukaemia. This work demonstrated how chemotherapy treatment promoted transient changes in glutamine metabolism, which could be therapeutically targeted with a broad inhibitor of glutamine metabolism, DON (a glutamine analogue) and a more specific inhibitor of the downstream pyrimidine synthesis pathway. Targeting this chemotherapy-imposed vulnerability improved response to chemotherapy and re-sensitised previously chemo-resistant residual AML cells [159], providing a strong proof of principal for imposing and sequentially targeting a common metabolic process in inherently heterogeneous tumour cells. This concept is represented in Figure 3, which graphically represents the phenomena of intrinsic and acquired resistance and how these can potentially be targeted through appropriate drug selection and scheduling.

Broad spectrum strategies such as restriction of glucose uptake through nutrient depletion or the use of glucose analogues such as 2-DG have shown promise in vitro, however the clinical application of such strategies proves challenging due to the almost ubiquitous requirement for glucose across most tissues and cell types (reviewed in [169]). Interest is now drawn towards the application of targeted agents to exploit innate and therapeutically-imposed metabolic vulnerabilities, such as those discussed above. A promising avenue to expedite this opportunity clinically is through repurposing of metabolic drugs currently applied to treat other conditions. Metformin, widely used for the treatment of diabetes due to its ability to inhibit hepatocellular gluconeogenesis, is an example of such an opportunity. Metformin also acts as an inhibitor of ETC complex-I [170], and therefore holds promise as a novel combination strategy with SoC therapies which select for cells with enhanced OXPHOS as a mechanism of resistance (as discussed above). As a single agent, used to treat diabetes in patients with cancer as a comorbidity, Metformin can reduce the incidence, mortality and relapse of cancers as well as improving outcomes in patients receiving SoC chemo/radiotherapies [171]. Hence, the molecular and clinical evidence advocating for treating cancers with an enriched OXPHOS phenotype with Metformin mono/combination therapy is robust and safe, and could be considered as a strategy to improve cancer patient outcomes.

### 1.6. Summary: Switching Metabolism from ‘Foe’ to ‘Friend’ of Cancer Therapy

We demonstrate herein the integral and powerful role the mitochondria play in maintaining cellular homeostasis, and how targeting or exploiting key functions of the mitochondria can revert this organelle from supportive *Friend* to lethal *Foe*. A wealth of drugs aimed at targeting mitochondrial systems such as BH3 mimetics and OXPHOS inhibitors are currently under investigation as potential anti-cancer agents and are at various stages of clinical development [172,173]. The development of BH3 mimetics such as the BCL-2 selective inhibitor Venetoclax has proven a success and is now approved for clinical management of a range of haematological malignancies, however other targeted BH3 mimetics have failed to succeed in this regard [174]. The mixed fortunes of translating BH3 mimetic in vitro to clinical efficacy is in part due to toxicity towards normal cells [174], but may also be due to the often-overlooked metabolic implications of therapeutically disrupting mitochondrial function [30,167]. Firstly, induction of partial-MOMP has the potential to further transform malignant cells rather than eliminate them [31] and secondly, treatment with some BH3 mimetics has been reported to increase intracellular ROS levels [175], which can be further transformative to the cell if at sub-toxic concentrations. Hence it is pertinent to consider using BH3 mimetics as an apoptosis-priming agent for complimentary therapies targeting metabolic pathways (and ROS management) proximal to the mitochondria. To support this theory, a number of research groups have shown that BH3 mimetics impose high levels of oxidative stress, and combined inhibition of antioxidant biosynthesis potently enhances the apoptotic effects [66,175]. Co-targeting metabolism and apoptosis by inhibiting both BCL-2 and OXPHOS complex-I has recently been proven a highly potent strategy to kill apoptosis-refractory and OXPHOS-reliant cancer cells [176,177]. In doing so, this combination strategy could force a catastrophic mitochondrial collapse with fully depolarised, permeabilised and metabolically defunct mitochondria. Importantly, clearance of such mitochondria should be both swift and efficient to eliminate the possibility of cancer cell persistence and propagation. Fundamental to ensuring only ‘healthy’ and fully functioning mitochondria exist within the cell are a range of quality control measures such as mitophagy and mito-nuclear communication to ensure the damaged, exhausted or sub-optimal mitochondrial are eliminated or repaired (expertly reviewed: [178]).

Therefore, targeting both the metabolic and apoptotic roles of the mitochondria in carefully selected patients, or those patients pre-treated with agents to force such metabolic dependencies could debilitate a ‘kingpin’ of cancer cell survival and truly convert the mitochondria and all its features from *Foe* to *Friend* of cancer therapy.

## Data Availability

Data sharing not applicable.
